# Parameter Sensitivity Study of the Johnson–Cook Model in FEM Turning of Ti6Al4V Alloy

**DOI:** 10.3390/ma18143351

**Published:** 2025-07-17

**Authors:** Piotr Löschner, Piotr Niesłony, Szymon Kołodziej

**Affiliations:** Faculty of Mechanical Engineering, Opole University of Technology, 76 Proszkowska St., 45-758 Opole, Poland; p.nieslony@po.edu.pl (P.N.); s.kolodziej@po.edu.pl (S.K.)

**Keywords:** Johnson–Cook model parameters, FEM simulation, Ti6Al4V, orthogonal turning

## Abstract

The aim of this study was to analyse in detail the effect of varying the parameters of the Johnson–Cook (JC) material model on the results of a numerical simulation of the orthogonal turning process of the Ti6Al4V titanium alloy. The first step involved an experimental study, including the recording of cutting force components and temperature, as well as the measurement of chip geometry, which was used to validate the FEM simulation. This was followed by a sensitivity analysis of the JC model with respect to five parameters, namely *A, B, C*, *m*, and *n*, each modified independently by ±20%. The effects of these changes on cutting forces, cutting zone temperature, stresses, and chip geometry were evaluated. The results showed that parameters *A, B*, and *m* had the greatest influence on the physical quantities analysed, while *C* and *n* are of secondary importance. The analysis highlighted the need for precise calibration of the JC model parameters, especially when modelling machining processes involving difficult-to-machine materials. The results provided practical guidance for optimising the selection of constitutive parameters in machining simulations.

## 1. Introduction

Modern machining processes, especially in high-tech sectors such as aerospace, automotive, and biomedical, are becoming increasingly demanding in terms of dimensional accuracy, surface quality, process predictability, and efficiency. The traditional experimental approach, based on numerous physical tests, is time-consuming and costly, especially considering the machining of difficult-to-machine materials such as titanium alloys. Consequently, computer-based modelling methods for machining processes are gaining increasing importance. Numerical simulations based on the finite element method (FEM) play a key role, as they enable the analysis of phenomena occurring in the cutting zone while reducing the number of experiments. Thanks to the development of computational tools and material databases, FEM has become an essential tool for the analysis and optimisation of machining operations. For example, Kandráč et al. [1] demonstrated its application for predicting cutting forces and chip formation during the orthogonal turning of titanium alloys. Lou et al. [2] used FEM for ultra-precision machining, focusing on achieving high surface quality. Song et al. [3] analysed pre-stress multi-step cutting, showing that FEM helps optimise residual stresses and thermal effects in complex machining processes.

The finite element method is widely used in the modelling of machining processes, enabling the detailed analysis of, among other things, stress distribution, displacement, temperature, and chip formation. However, a key aspect of the accuracy of such simulations remains the selection of an appropriate material model, which must adequately represent the behaviour of the material over a wide temperature range and at high strain rates [4,5]. Improperly chosen constitutive parameters can lead to significant errors in predicting cutting forces, chip geometry, or temperature in the cutting zone. This aspect is particularly important for titanium alloys such as Ti6Al4V, which are widely used but very challenging to machine due to their low thermal conductivity, high strength at elevated temperatures, and tendency to cause rapid tool wear. These characteristics make reliable FEM simulation an essential tool for process optimisation and prediction when machining Ti6Al4V. In recent years, there has been growing interest in modern constitutive models that go beyond classical empirical approaches. In addition to simple phenomenological models such as Johnson–Cook, microstructure-based models [6], damage models with fracture criteria [7], and hybrid approaches combining experimental data with inverse modelling [8,9] are increasingly used. In recent years, neural networks have increasingly been used as alternative or hybrid constitutive models. They can act as universal function approximators to predict complex stress–strain relationships directly from the data, or be combined with physical constraints in physics-informed neural networks (PINNs). Such approaches can improve prediction accuracy for materials with complex behaviours, but require extensive datasets for training and careful validation [10].

The Johnson–Cook model is one of the most widely used phenomenological material models for describing the behaviour of a material under high plastic deformation, elevated temperature, and significant strain rates. It accounts for three main mechanisms: strain hardening, strain rate effects, and thermal softening. Mathematically, it is expressed as a multiplicative function of five parameters: *A* (initial yield stress), *B* (strain hardening modulus), *C* (strain rate sensitivity coefficient), *m* (thermal softening exponent), and *n* (strain hardening exponent). A key advantage of the model is that it can be calibrated using conventional tensile tests and dynamic Hopkinson bar experiments [11,12]. The JC model is highly effective in representing dynamic processes, but it does not account for microstructural phenomena, dynamic recrystallisation, or strain localisation, which limits its accuracy. Therefore, modified versions of the model are being developed [13].

The parameters of the Johnson–Cook model can be determined through classical uniaxial tests (tension, compression) or by using dynamic Hopkinson bar experiments. However, alternative methods involving the calibration of model parameters based on data from actual cutting processes and the application of various optimisation algorithms are increasingly being adopted [14]. Although these approaches provide a better fit to real-world conditions, they often result in significant discrepancies in the parameter values reported by different authors. Consequently, sensitivity analyses and the evaluation of the influence of each parameter on the final results of FEM simulations are becoming increasingly important [4,15,16].

Numerous research papers attempt to quantify the influence of individual Johnson–Cook model parameters on FEM simulation results. Parameter *A* is responsible for the initial level of flow stress. Its increase leads directly to higher cutting forces and alters heat distribution in the cutting zone. Shen et al. [15] demonstrated that parameter *A* has a strong influence on the maximum cutting force. Parameters *B* and *n*, on the other hand, describe the effect of material hardening. Their increase leads to greater resistance to plastic deformation, which affects the resulting chip morphology. Shen et al. [15] observed that higher values of parameter *n* favour chip segmentation, while Zhu et al. [17] confirmed this effect in the context of high-temperature drilling of Ti6Al4V alloy. Gerstgrasser et al. [11], in turn, showed that changes in *B* and *n* significantly affect the accuracy of crack growth predictions in the workpiece material. Parameter *C* determines the material’s sensitivity to strain rate. A study by Zhu et al. [17] showed that a higher value of *C* results in a local increase in temperature at the tool–material interface and leads to more intensive tool wear. Parameter *m*, related to thermal softening, controls the extent to which flow stress decreases with increasing temperature. Thornton et al. [18] noted that underestimating this parameter leads to inaccurate chip segmentation modelling and can cause non-linear fluctuations in machining forces. The literature also highlights the existence of non-linear interactions between parameters—for example, simultaneous increases in *B* and *m* can produce complex synergistic effects that are difficult to predict without a full sensitivity analysis. Shen et al. [15] and Wang et al. [19] compared multiple sets of JC parameters and concluded that combinations of parameters have a greater effect on cutting forces than variations in individual values. Consequently, modern methods are increasingly used to identify the most influential parameter combinations [5,16,20].

Despite the many papers analysing the JC model, there is still a gap in the literature regarding a systematic and isolated analysis of the effect of each of its parameters on key physical variables in turning simulations of difficult-to-machine alloys such as Ti6Al4V. Previous studies often focused on combined effects or used parameter sets without detailed sensitivity evaluation. This study addresses this gap by providing an individual parameter sensitivity analysis for the orthogonal turning of Ti6Al4V, focusing on the influence of *A*, *B*, *C*, *m*, and *n* on cutting forces, temperature distribution, and chip geometry. This new insight can help improve the precision of JC model calibration for machining simulations of titanium alloys.

## 2. Verification of the Johnson–Cook Material Model

### 2.1. Experimental Studies

The aim of the experimental study was to compare and numerically validate the Johnson–Cook material model for orthogonal turning of the Ti6Al4V titanium alloy. The validation of the finite element method (FEM) simulations was primarily based on the analysis of cutting force components, the maximum temperature in the cutting zone, and the dimensional and geometric characteristics of the chip.

**Figure 1:**
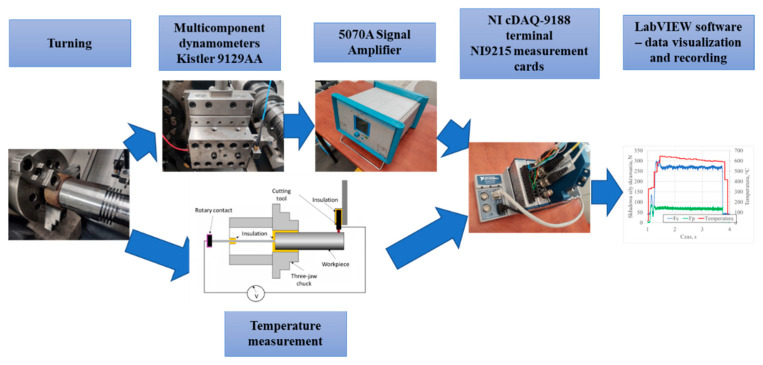
Schematic of the measurement setup for recording cutting force components and temperature using the natural thermocouple method.

The experiments were conducted on a multifunctional 3-axis CNC lathe (Okuma Genos L200E-M), equipped with a measurement setup capable of recording the Cartesian components of cutting forces. The cutting forces were measured using a piezoelectric dynamometer (Kistler model 9129AA), while the temperature in the cutting zone was measured using the natural thermocouple method. The calibration of the measurement system and the related accuracy have been described in detail in [21]. A schematic of the measurement setup is shown in Figure 1.

Experimental studies of orthogonal turning were performed using cutting tools manufactured by Tungaloy Corporation (Iwaki, Japan). A carbide insert for orthogonal turning, designated DGG500-0400, was used in the experiments. The insert was clamped in a CTER2020-5T12 toolholder, which allowed the following angles to be obtained in the working system:

- Orthogonal rake angle γₒ = 20°;
- Normal clearance angle αₒ = 7°.

The experiments were performed under dry cutting conditions, without the use of coolant, at an ambient temperature of approximately 20 °C. The test matrix included six cutting speeds (*v_c_* = 40, 50, 60, 70, 80, and 90 m/min), three feed rates (*f* = 0.05, 0.1, and 0.15 mm/rev), and a constant depth of cut *a_p_* = 3 mm.

### 2.2. Simulation Studies

Simulation studies of the orthogonal turning process of the Ti6Al4V titanium alloy were conducted using the same technological parameters as in the experimental studies. The aim of the research was to verify the accuracy of the material parameter identification for the Johnson–Cook model. The procedure for determining the individual parameters of the JC model was presented in [21]. Using an analytical method, the following Johnson–Cook parameters were obtained, as listed in Table 1**.**

The Design Environment for Forming (DEFORM 2D/3D) engineering simulation system, developed by Scientific Forming Technologies Corporation, was used for the simulation studies. FEM simulations were performed in the DEFORM v12.0.2 environment, using the predefined material parameters of the Johnson–Cook model.

The mechanical, tribological, and thermophysical properties of the workpiece material were defined as temperature-dependent using data from the DEFORM 2D/3D material library. The simulation used an adaptive mesh with quadrilateral elements, with local remeshing in the cutting zone to maintain accuracy. Boundary conditions were set to fully fix the workpiece at the rear side, while the tool was modelled as rigid. Friction at the tool–chip interface was described by the Coulomb friction model with a coefficient of µ = 0.7. The technological parameters in the first stage of the simulation matched the experimental tests, enabling direct comparison.

### 2.3. Verification of Simulation Studies

**Figure 2:**
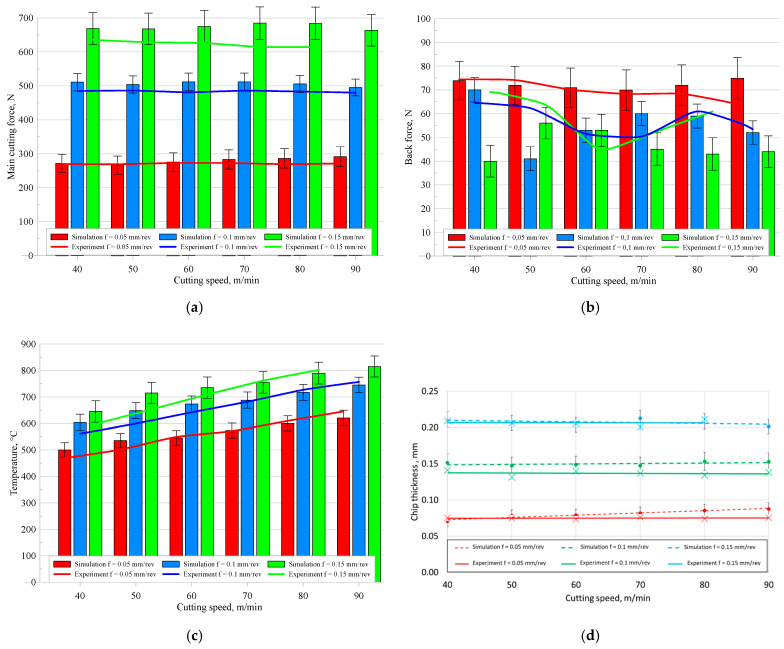
Comparison of simulation and experimental results for (**a**) main cutting force, (**b**) back cutting force, (**c**) cutting zone temperature, and (**d**) chip thickness.

After the simulation and experimental studies, a comparative analysis of the obtained results was conducted. Figure 2 presents a comparison between the numerical simulation results and the data obtained from the experimental measurements. Due to catastrophic tool wear during tests with a feed rate of *f* = 0.15 mm/rev and a cutting speed of *v_c_* = 90 m/min, it was not possible to obtain repeatable experimental data for this parameter combination. The detailed test procedure and results for this specific case are provided in our previous publication [21].

The comparison between the numerical simulation and experimental results focused on three aspects: the cutting force components, the temperature in the cutting zone, and the geometry and thickness of the chip. The main cutting force component *F_c_* was reproduced with high accuracy—the differences between the experimental and simulation results in most cases did not exceed 8%, and were less than 2% at low feed rates. Larger discrepancies were observed for the back force *F_p_* especially at high feed rates, where deviations reached up to 42%. This can be explained by the fact that in orthogonal cutting the back force component is typically very small and highly sensitive to minor tool vibrations, slight misalignments, and local tool wear, which are difficult to reproduce accurately in a 2D simulation. The temperature analysis showed a consistency of 3–4%, regardless of the cutting parameters used, confirming the correct representation of thermal phenomena. The shape and thickness of the chips also showed good agreement—the largest differences in chip thickness did not exceed 14%, with the best fit observed at the highest feed rate. The obtained results confirm that the identified parameters of the JC model enable a reliable representation of the actual course of the Ti6Al4V turning process and can be effectively used in numerical analyses within the tested range of technological parameters. After finding satisfactory agreement between the FEM simulation results and experimental data, it was concluded that the determined Johnson–Cook model parameters reliably represent the phenomena occurring in the cutting zone during orthogonal turning of the Ti6Al4V alloy. In the next stage of the research, a detailed analysis of the influence of variations in individual JC model parameters on selected physical characteristics of the process was performed. The aim of this analysis was to assess the model’s sensitivity to changes in material parameters and to identify the parameters that most significantly influence the cutting process.

## 3. Evaluation of the Influence of Changes in the Parameters of the Johnson–Cook Constitutive Model on the Physical Characteristics of the Cutting Process

A sensitivity analysis of the Johnson–Cook model was conducted to examine how modifications to individual parameters (*A*, *B*, *C*, *m*, *n*) affect physical quantities of the turning process, such as cutting forces (*F_c_*, *F_p_*), temperature in the cutting zone, material stresses, and chip characteristics. Each parameter was independently increased and decreased by 20% relative to the baseline value, while the other model parameters were kept constant. This approach allowed the relative influence of each parameter on the individual variables to be determined and enabled the identification of those with the greatest relevance to the accuracy of cutting process prediction. The model names clearly indicate which parameter was modified and by how much—for example, **“***A* +20%” refers to the model in which parameter *A* was increased by 20% relative to the baseline. Table 2 presents the values of the material parameters used in the analysis, including both the baseline model and the modified variants.

### 3.1. Stress–Strain Curves

**Figure 3:**
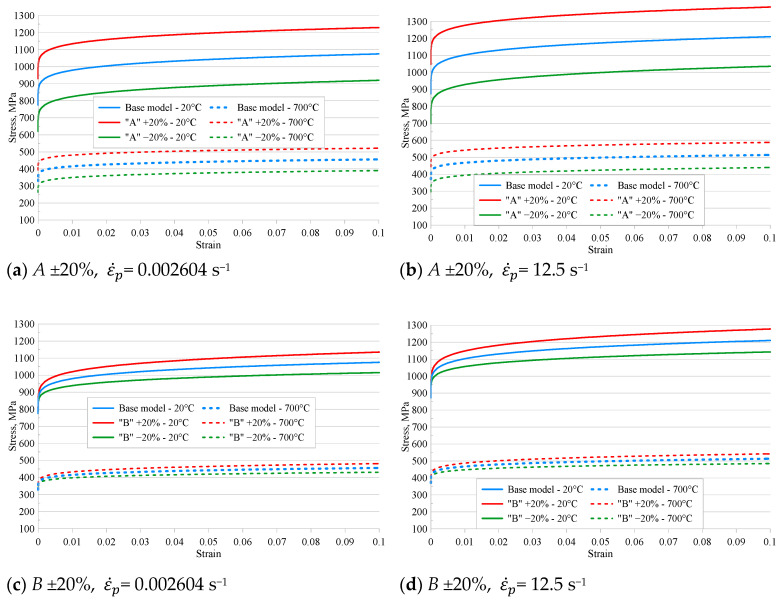

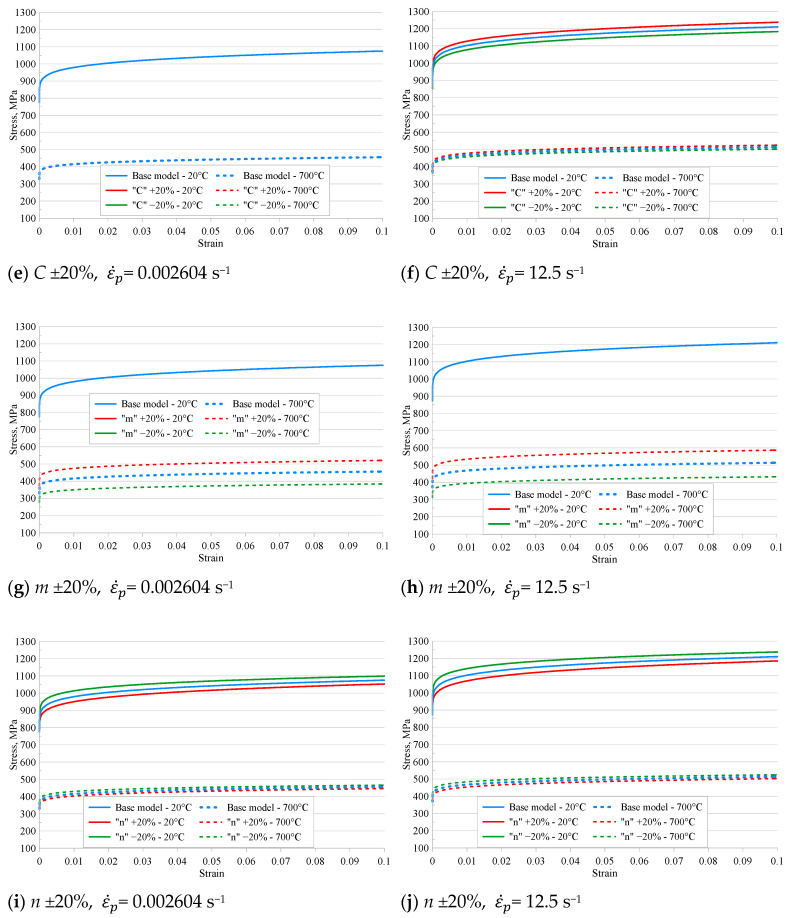
Effect of variations in individual parameters of the Johnson–Cook constitutive model on the stress–strain curve at strain rates of 0.002604 s^−1^ and 12.5 s^−1^.

Performing a stress–strain analysis is a key step in assessing the behaviour of a material under real-world cutting process conditions. To determine the influence of individual parameters of the Johnson–Cook constitutive model on the material response, an analysis of the σ–ε curves was performed for different model variants. The analysis was conducted for two representative strain rates: one low and one high. This approach made it possible to capture differences resulting from both strain rate sensitivity and thermal softening, which are incorporated into the structure of the JC model. These temperatures were chosen as they represent the ambient laboratory temperature (20 °C) and the estimated maximum cutting zone temperature (700 °C) for Ti6Al4V during orthogonal turning. The aim of this analysis was to identify the parameters that most significantly influence the value and shape of the stress–strain curve. The following section presents the results and a detailed interpretation of the effects of parameter variations on the σ–ε curves. Figure 3 presents the stress–strain curves for the modified Johnson–Cook material models at two strain rates: 0.002604 s^−1^ and 12.5 s^−1^. The base curve was generated by fitting the experimental tensile and compression test data using the MATLAB R2019b Curve Fitting Tool, as described in [21]. The additional curves illustrate the effect of increasing or decreasing each Johnson–Cook parameter (*A, B, C, m, n*) by ±20% to analyse the parameter sensitivity.

When analysing the effect of varying individual Johnson–Cook model parameters on the stress–strain curve, it was noted that the most significant impact on stress values was observed for parameter *A*. Increasing or decreasing the value of *A* by 20% resulted in a change in stress at a given strain of approximately ±155 MPa at 20 °C and ±66 MPa at 700 °C for low strain rates. A similar pattern of variation was observed at higher strain rates, where the stress difference reached ±175 MPa at 20 °C and ±74 MPa at 700 °C. This indicates that parameter *A* plays a key role in determining the level of initial (incipient) stress, and modifying it proportionally shifts the entire stress–strain curve vertically.

Parameter *B*, which governs strain hardening, exhibited a lesser influence on the stress–strain curve. As the strain increased, the differences between the baseline and modified models became more pronounced. The maximum difference observed within the tested parameter range was ±60 MPa (±5.6%) at a strain of 0.1 and 20 °C, and ±29 MPa (±5.6%) at 700 °C under low-strain-rate conditions. Comparable differences were observed at higher strain rates, where the maximum difference increased to ±68 MPa (±5.6%), while for 700 °C the same level of relative strain change (±5.6%) was maintained. The influence of parameter *B* becomes more pronounced at higher strain rates, where the material undergoes intense hardening.

Modifying the strain rate sensitivity coefficient *C* had little effect on the curve under low-strain-rate conditions, since the reference and actual strain rates were equal, resulting in no stress change. As the strain rate increased, larger deviations between the curves of the modified models were observed; however, the overall effect remained relatively small. The maximum difference recorded was ±2%, which confirms the logarithmic nature of strain rate effects in the Johnson–Cook model.

The strongest temperature-dependent influence on stress values was associated with parameter *m*, which controls thermal softening. At a reference temperature of 20 °C, variations in *m* had no effect on the stress level, which is consistent with the definition of the Johnson–Cook model. However, as temperature increased, significant deviations from the baseline model were observed. At 700 °C, stress differences ranged from –14.2% to +15.8% under low-strain-rate conditions, indicating that this parameter significantly affects material softening at elevated temperatures. Similar behaviour was observed for higher strain rates.

The final parameter analysed was the strain hardening exponent *n*, which controls the rate of stress increase as a function of strain. The variation of “*A* ±20%” in the value of *n* resulted in stress differences ranging from –4% to +3.1%, confirming its role in defining the intensity of material strengthening. Increasing *n* led to a decrease in stress relative to the baseline model, whereas decreasing *n* resulted in a stress increase. This effect was visible across the entire temperature and strain rate range but had little impact on the initial stress values.

This study confirms that the sensitivity of the Johnson–Cook model to parameter variation depends on the loading conditions. Parameters *A* and *B* have the greatest influence on stress–strain behaviour, while *C* and *n* exhibit a lesser effect. Parameter *m* is particularly important when analysing materials subjected to elevated temperatures.

### 3.2. Chip Characteristics

**Figure 4:**
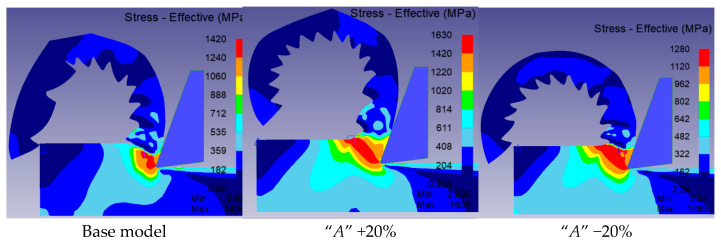

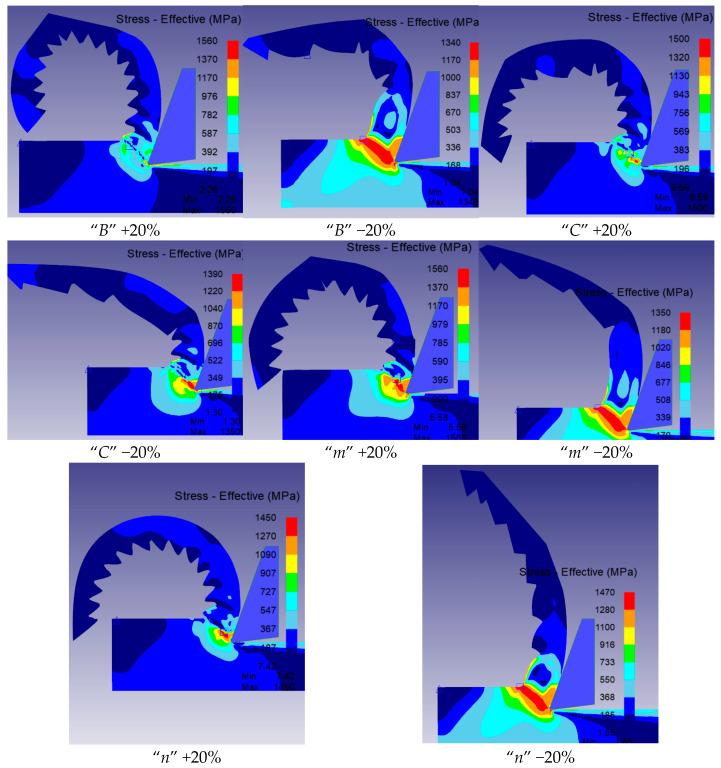
Chip shapes and geometries obtained in simulations using modified parameters of the Johnson–Cook model.

The first step in evaluating the FEM simulation results was a visual assessment of the chip formed during orthogonal cutting, followed by the measurement of its characteristic dimensions. Figure 4 presents the chip shapes obtained for the baseline model and its parameter modifications. Table 3 presents the characteristic dimensions of the chips.

The analysis of the effect of varying individual parameters of the Johnson–Cook constitutive model on the chip characteristics included both a visual assessment of the chip shape and the measurement of its characteristic dimensions. Based on Figure 4 and the data in Table 3**,** it can be observed that each modification of the model parameters affects the chip geometry and its regularity to varying degrees.

When evaluating the influence of individual Johnson–Cook model parameters on chip shape, it was found that parameter *A* has the least impact. *A* 20% decrease in the value of *A* results in a maximum change of 8% in the characteristic chip dimensions. In contrast, a 20% increase in *A* causes a 12.5% change in those dimensions.

A further analysis revealed that increasing the remaining parameters by 20% generally results in a sawtooth chip shape, whereas decreasing these parameters causes the loss of the characteristic sawtooth profile. As a consequence, the vertex distance measured on the chip’s outer surface increases. When the parameters were decreased by 20%, the vertex distance increased by 121% to 213% compared to the baseline model, demonstrating a strong influence of these parameters on the chip geometry in FEM simulations. In contrast, increasing the parameters by 20% resulted in a maximum difference of only 8%.

It was also observed that changes in the individual parameters had no significant impact on chip thickness at the tooth tips. For the assumed range of parameter variations, the maximum deviation from the baseline model reached 8.5%. *A* similar trend was noted when analysing the chip thickness at the valleys: parameter changes of ±20% resulted in a maximum variation of ±9% in thickness relative to the baseline model.

### 3.3. Cutting Forces

**Figure 5:**
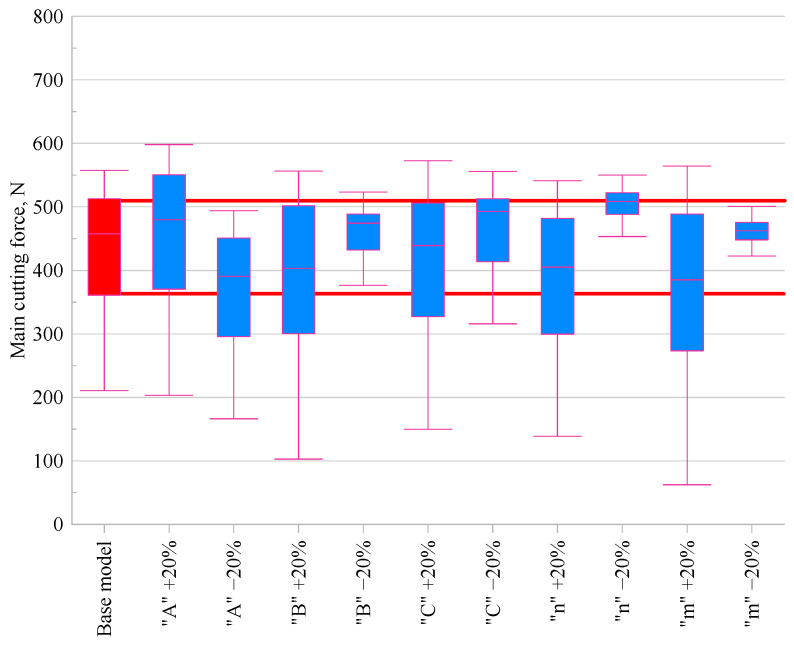
Comparison of main cutting force *F_c_* values for modified Johnson–Cook material models.

**Figure 6:**
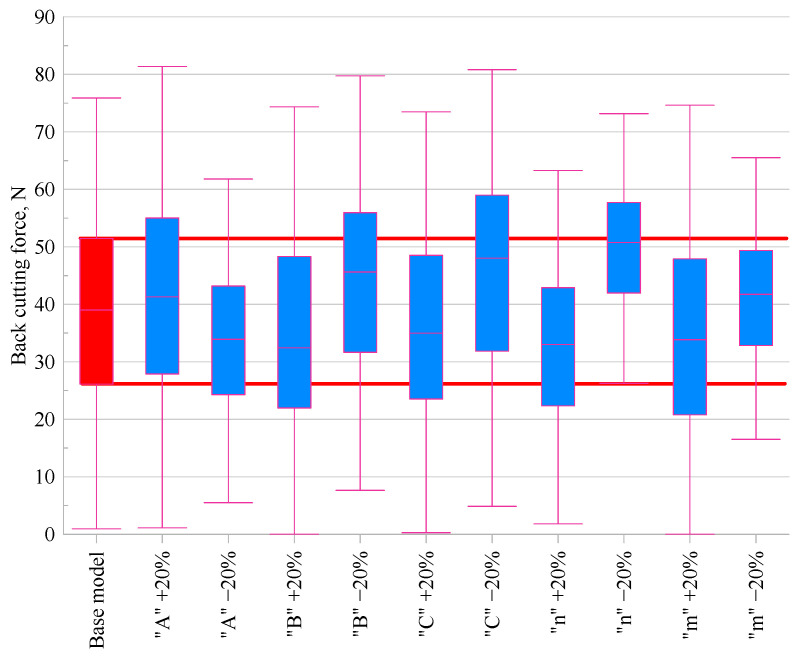
Comparison of back cutting force *F_p_* values for modified Johnson–Cook material models.

In order to compare the effect of changing individual parameters of the Johnson–Cook model, the variations in the main cutting force (*F_c_*) and back force (*F_p_*) were evaluated relative to the base model. Figure 5 and Figure 6 present the results in the form of box plots.

An analysis of the effect of varying individual material constants of the Johnson–Cook model on the values of the main cutting force (*F_c_*) and the back force (*F_p_*) is presented based on the data from the graphs. The analysis was conducted relative to the base model, for which the median values were *F_c_* = 458 N and *F_p_* = 39 N.

An analysis of Figure 5**,** which illustrates the effect of parameter variation on the main cutting force, revealed that modifying the *A* parameter of the Johnson–Cook model by ±20% had a significant impact on *F_c_*. Increasing *A* raised the force to 480 N (+4.8%), whereas decreasing *A* reduced it to 391 N (–14.6%) relative to the base model. This is because parameter *A* directly represents the initial flow stress level, and therefore its increase raises the overall cutting resistance of the material, which increases the cutting forces. Parameter *B* also showed a marked effect: increasing *B* resulted in a reduced force of 403 N (–11.9%), while decreasing *B* raised *F_c_* to 474 N (+3.6%). For parameter *C*, the influence was noticeable but less pronounced. Increasing *C* reduced the cutting force to 439 N (–4.0%), whereas decreasing it increased the force to 493 N (+7.7%), indicating a moderate sensitivity of the force to strain rate effects. The influence of the *n* parameter, associated with strain hardening, showed an asymmetric response, increasing *n* reduced *F_c_* to 405 N (–11.5%), while decreasing it elevated the force to 509 N (+11.1%). Finally, parameter *m*, which accounts for thermal softening, had a moderate effect. Decreasing *m* increased *F_c_* to 463 N (+1.1%), while increasing it led to a more substantial reduction to 385 N (–15.9%). Parameter *m* affects the extent of stress reduction with increasing temperature, which also influences the cutting force level.

An analysis of Figure 6, which illustrates the effect of changing the Johnson–Cook model parameters on the back cutting force (*F_p_*), showed that increasing *A* raised the force to 41.3 N (+5.9%), while decreasing it reduced the force to 34.0 N (–13.0%). For parameter *B*, a significant asymmetry in its influence was observed: increasing *B* decreased *F_p_* to 32.5 N (–16.8%), whereas decreasing *B* increased the force to 45.6 N (+17.0%). Changes in parameter *C* also led to noticeable variations: increasing *C* reduced *F_p_* to 35.0 N (–10.4%), while decreasing it raised the value to 48.0 N (+23.1%). Parameter *n* exhibited the most pronounced influence on the back force. Increasing *n* reduced *F_p_* to 33.0 N (–15.4%), whereas decreasing *n* increased it substantially to 50.8 N (+30.1%), confirming its key role in shaping the material’s plastic flow characteristics. As in the case of the main cutting force, parameter *m* had a moderate effect: decreasing *m* led to an increase in *F_p_* to 41.8 N (+7.0%), while increasing it reduced the force to 33.8 N (–13.3%).

### 3.4. Temperature in the Cutting Zone

**Figure 7:**
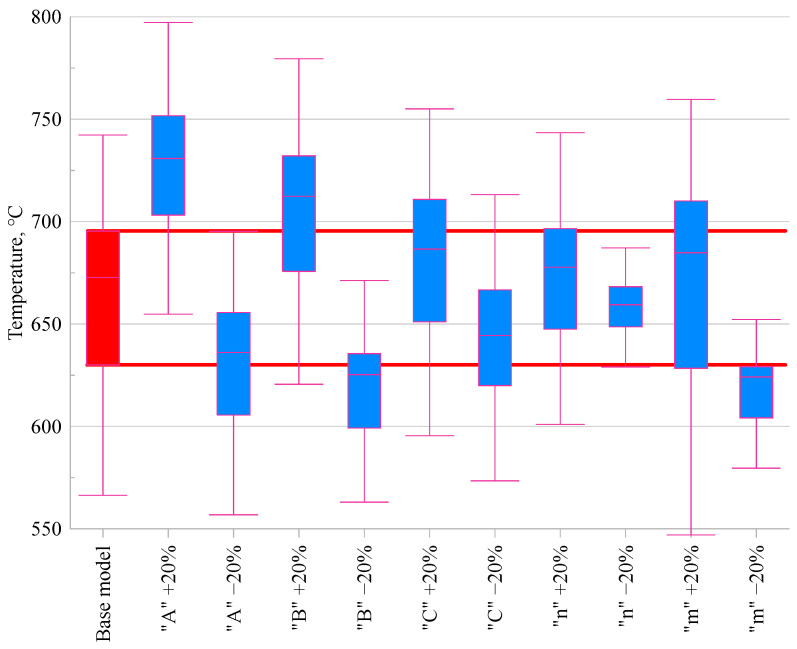
Comparison of the maximum temperature value occurring in the cutting zone for modified Johnson–Cook constitutive models.

In the next stage of the analysis, the effect of changes in the Johnson–Cook model parameters on the temperature in the cutting zone was evaluated. Figure 7 presents the results of the FEM simulations, enabling a comparison of temperature values obtained for the base model and for variants in which the parameter values were altered by ±20%.

When analysing the temperature in the cutting zone (Figure 7)**,** it can be observed that the parameters *A*, *B*, and *m* exert the greatest influence on the temperature value. An increase in the initial flow stress (*A*) leads to a temperature rise of +8.6%**,** while its decrease results in a –5.5% reduction. A similar effect is noted for parameter *B*, with an increase causing a +5.8% rise and a decrease resulting in a –7.1% drop, indicating its significant contribution to the intensification of thermal effects associated with material hardening. This increase can be explained by the fact that the higher flow stress resulting from larger *A* and *B* values leads to greater plastic deformation work, which is largely converted into heat in the cutting zone. In the case of parameter *m*, which governs thermal softening, variations also lead to noticeable changes: −7.3% for a decrease and +1.8% for an increase. The strain rate sensitivity coefficient *C* produced relatively minor differences—within ±2%—confirming its limited effect on thermal behaviour in this context. Similarly, modifications of parameter *n* (strain hardening exponent) had only a marginal influence on cutting temperature, with variations of less than ±2.1%.

### 3.5. Stress in the Material

**Figure 8:**
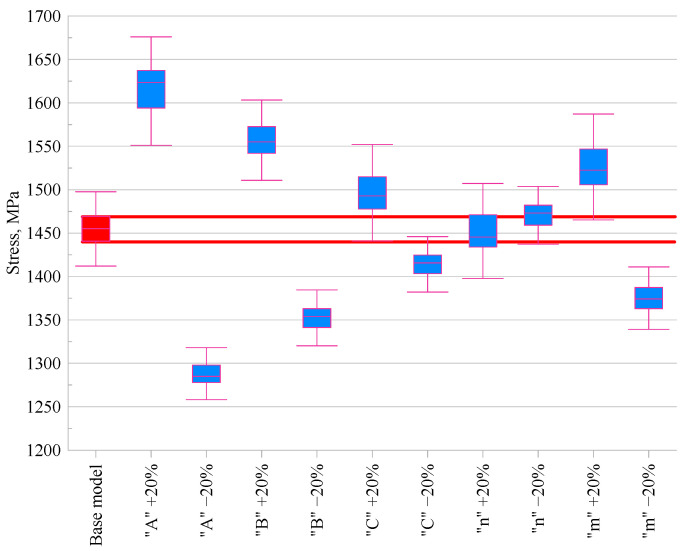
Comparison of the maximum stress occurring in the material for modified Johnson–Cook constitutive models.

The effect of changing the values of the material constants in the Johnson–Cook constitutive model was also assessed in relation to the stresses in the material. Figure 8 showed the impact of modifying individual constants on the stress results obtained.

Analysing Figure 8, which presents the effect of changing the JC model parameters on the stress in the material, it can be observed that parameter *A*, representing the yield strength at the reference temperature, has the greatest influence on the stress value. An increase in this parameter led to an 11.6% rise in stress, while a decrease resulted in an 11.7% reduction, clearly indicating that *A* plays a key role in determining the stress level. Changes in parameter *B*, responsible for material hardening, showed a similar but slightly less pronounced effect, with an increase in *B* leading to a 6.9% increase in stress and a decrease resulting in a reduction of the same magnitude. For parameters *C*, *m*, and *n*, the observed changes were less significant. The influence of *C* and *m* remained within ±2.7%, which is consistent with their less dominant role in stress development. For parameter *n*, the differences were minimal, reaching up to ±1.2%, indicating its more subtle effect on material strengthening under the conditions analysed. However, it should be noted that the Johnson–Cook model does not capture microstructural phenomena such as dynamic recrystallisation, which may influence stress predictions at high temperatures. More advanced physically based models exist, but they require extensive microstructural data and calibration, which were beyond the scope of this sensitivity study.

## 4. Conclusions

In this study, a comprehensive analysis was performed to investigate the influence that the Johnson–Cook constitutive model parameters have on key characteristics of the cutting process of titanium alloy Ti6Al4V, such as cutting forces, temperature in the machining zone, stresses, and chip geometry. As a first step, the numerical model was validated using experimental data obtained from an actual orthogonal turning process. The simulation results showed very good agreement with the experimental measurements, especially in terms of cutting forces and chip thickness, which confirmed the correctness of the material parameters used in the base model.

The remainder of the paper focused on assessing the sensitivity of the JC model to changes in individual parameters: *A*, *B*, *C*, *m*, and *n*. The analysis showed that

- the *A* parameter (plasticising stress) has the greatest influence on the level of cutting forces, temperature, and stresses;
- the *B* parameter, which is responsible for the strengthening effect of the material, significantly shapes the distribution of stresses and temperatures in the cutting zone and the chip, and its effect on forces is moderate;
- the *C* parameter has a moderate effect on all the characteristics analysed, which is consistent with the logarithmic nature of its dependence on strain rate;
- the *m* parameter, which describes thermal softening, is most influenced by temperatures in the cutting zone and stress;
- the *n* parameter, related to plastic strengthening, mainly affects the components of the cutting force, with a marginal effect on other aspects.

These observations suggest that effective calibration of the Johnson–Cook model parameters requires special attention to the parameters *A*, *B*, and *m*, due to their dominant influence on the thermomechanical phenomena in the cutting process. The use of models based on the literature and unverified parameter values can lead to significant prediction errors in FEM simulations.

## Figures and Tables

**Table 1:** Material constants of the Johnson–Cook model determined by the analytical method [21].

*A*	*B*	*C*	*n*	*m*
775.2	440.3	0.01483	0.1668	0.6269

**Table 2:** Sets of parameter values for the Johnson–Cook model.

Material Model	*A*	*B*	*C*	*m*	*n*
Base model	775	440	0.01483	0.6269	0.1668
“*A*” +20%	**930**	440	0.01483	0.6269	0.1668
“*A*” -20%	**620**	440	0.01483	0.6269	0.1668
“*B*” +20%	775	**528**	0.01483	0.6269	0.1668
“*B*” −20%	775	**352**	0.01483	0.6269	0.1668
“*C*” +20%	775	440	**0.01780**	0.6269	0.1668
“*C*” −20%	775	440	**0.01186**	0.6269	0.1668
“*m*” +20%	775	440	0.01483	**0.7523**	0.1668
“*m*” −20%	775	440	0.01483	**0.5015**	0.1668
“*n*” +20%	775	440	0.01483	0.6269	**0.2002**
“*n*” −20%	775	440	0.01483	0.6269	**0.1334**

**Table 3:** Average values of characteristic chip dimensions for modified parameters of the Johnson–Cook constitutive material model.

Material Model	Chip Thickness on Top, mm	Chip Thickness in the Valley, mm	Chip Tip Distance, mm
Base model	**0.153**	**0.102**	**0.063**
“*A*” +20%	0.134	0.089	0.058
“*A*” −20%	0.155	0.106	0.068
“*B*” +20%	0.140	0.095	0.060
“*B*” −20%	0.154	0.096	0.178
“*C*” +20%	0.143	0.098	0.059
“*C*” -20%	0.150	0.1	0.196
“*m*” +20%	0.145	0.098	0.063
“*m*” −20%	0.144	0.111	0.197
“*n*” +20%	0.149	0.103	0.059
“*n*” −20%	0.145	0.104	0.139

## Data Availability

The original contributions presented in this study are included in the article. Further inquiries can be directed to the corresponding author.
